# Haplotype blocks for genomic prediction: a comparative evaluation in multiple crop datasets

**DOI:** 10.3389/fpls.2023.1217589

**Published:** 2023-09-05

**Authors:** Sven E. Weber, Matthias Frisch, Rod J. Snowdon, Kai P. Voss-Fels

**Affiliations:** ^1^ Department of Plant Breeding, Justus Liebig University, Giessen, Germany; ^2^ Department of Biometry and Population Genetics, Justus Liebig University, Giessen, Germany; ^3^ Institute for Grapevine Breeding, Hochschule Geisenheim University, Geisenheim, Germany

**Keywords:** genomic selection, SNP markers, haploblocks, haplotype blocks, genomic prediction

## Abstract

In modern plant breeding, genomic selection is becoming the gold standard for selection of superior genotypes. The basis for genomic prediction models is a set of phenotyped lines along with their genotypic profile. With high marker density and linkage disequilibrium (LD) between markers, genotype data in breeding populations tends to exhibit considerable redundancy. Therefore, interest is growing in the use of haplotype blocks to overcome redundancy by summarizing co-inherited features. Moreover, haplotype blocks can help to capture local epistasis caused by interacting loci. Here, we compared genomic prediction methods that either used single SNPs or haplotype blocks with regards to their prediction accuracy for important traits in crop datasets. We used four published datasets from canola, maize, wheat and soybean. Different approaches to construct haplotype blocks were compared, including blocks based on LD, physical distance, number of adjacent markers and the algorithms implemented in the software “*Haploview*” and *“HaploBlocker”*. The tested prediction methods included Genomic Best Linear Unbiased Prediction (GBLUP), Extended GBLUP to account for additive by additive epistasis (EGBLUP), Bayesian LASSO and Reproducing Kernel Hilbert Space (RKHS) regression. We found improved prediction accuracy in some traits when using haplotype blocks compared to SNP-based predictions, however the magnitude of improvement was very trait- and model-specific. Especially in settings with low marker density, haplotype blocks can improve genomic prediction accuracy. In most cases, physically large haplotype blocks yielded a strong decrease in prediction accuracy. Especially when prediction accuracy varies greatly across different prediction models, prediction based on haplotype blocks can improve prediction accuracy of underperforming models. However, there is no “best” method to build haplotype blocks, since prediction accuracy varied considerably across methods and traits. Hence, criteria used to define haplotype blocks should not be viewed as fixed biological parameters, but rather as hyperparameters that need to be adjusted for every dataset.

## 1 Introduction

Genomic prediction has greatly improved animal and plant breeding (Hickey et al., 2017) and has the potential to improve genetic gain even in crops with complex genomes (Voss-Fels et al., 2021). In the past, predictions based on linear mixed models used relatedness to borrow information on target phenotypes of relatives. Henderson (1975) derived this relationship from pedigrees *via* the numerator relationship matrix with the expectation that each parent contributes exactly 50% of its genome to its offspring. With the advance of sequencing technology nowadays, genomic data is used to replace the pedigree relationship with realized relationships calculated from dense marker maps. Furthermore, with the inclusion of genetic markers, information about linkage disequilibrium and cosegregation is available for genomic prediction (Habier et al., 2013). Today, individuals in breeding populations of major crops can be sequenced with high quality at low costs, enabling the identification of millions of genome-wide single nucleotide polymorphism (SNP) markers that can be easily screened in large populations using high-throughput genotyping technologies. Together with phenotype measurements, genome-wide marker profiles can be used to predict breeding values of non-phenotyped individuals (Lande and Thompson, 1990; Bernardo, 1994; Meuwissen et al., 2001; VanRaden, 2008). This can assist breeders in the accurate identification of superior genotypes within their breeding material without the need for additional phenotyping. Moreover, it can facilitate the decision-making process for selecting which genotypes should undergo phenotyping, leading to reduced phenotyping costs and improved accuracy in estimating breeding values. Hence, genomic selection has the potential to considerably increase genetic gain and profit in many crops (Voss-Fels et al., 2021).

There are a variety of statistical methods for genome-based predictions (e.g. VanRaden, 2008; de los Campos et al., 2009; Zhang et al., 2010; Gianola, 2013; Hofheinz and Frisch, 2014; Werner et al., 2018a; Millet et al., 2019), differing in their assumptions of variance components, marker effects or marker modes of action. Examples for genomic prediction models are ridge regression BLUP, GBLUP (Bernardo, 1994; Meuwissen et al., 2001; VanRaden, 2008), Reproducing Kernel Hilbert Space Regression (RKHS) (de los Campos et al., 2009), as well as Bayesian models like Bayesian LASSO (Park and Casella, 2008) or Bayesian ridge regression (Pérez and de los Campos, 2014).

However, biallelic SNPs are sometimes unable to identify all variants and allelic combinations of genes that contribute to a particular trait, since most genes carry multiple sequence polymorphisms. Furthermore, accurate genomic prediction is often obtained based on close relatives (VanRaden, 2008; Hayes et al., 2009) while this accuracy decreases as the validation individuals get more unrelated (Habier et al., 2010; Wolc et al., 2011). This implies that SNPs are not necessarily in LD with causal QTL and the prediction accuracy is at least partly driven by implicitly capturing relationship among individuals. Hence, one strategy to improve predictions is increasing marker density. With the advance of whole genome sequencing technologies, increasingly large and dense marker datasets can today be generated for most major crops (Edwards and Batley, 2010; Yu et al., 2011). However, increasing marker density does not consistently improve prediction accuracies (Solberg et al., 2008; Druet et al., 2014; Hayes et al., 2014; Norman et al., 2018) and often improvements are only observed following pre-selection of markers (van Binsbergen et al., 2015; Ni et al., 2017; Raymond et al., 2018). Furthermore, prediction accuracy is influenced by trait heritability (Zhang et al., 2017) and the number of genotypes with phenotypic records available for genomic selection. Hence, another approach to enhance prediction accuracy is by increasing the number of phenotyped lines used for model training (VanRaden et al., 2009; Combs and Bernardo, 2013). However, due to the high costs associated with phenotyping, this may not always be feasible, particularly when sparse testing methods (Jarquin et al., 2020; Crespo-Herrera et al., 2021; Atanda et al., 2022; Terraillon et al., 2022) are not applicable. Hence, one strategy to address low prediction accuracy could be to identify more informative variants for predictions without necessarily increasing the marker density *per se*.

Loci along the genome are usually inherited in a block-like structure, with only few recombination hotspots (Daly et al., 2001; Jeffreys et al., 2001; Reich et al., 2001) defining the so-called haplotype blocks. There are several ways to define a haplotype block, for example as a fixed window of adjacent markers, as a fixed window of adjacent base pairs, or based on a statistical measure of LD. While the first two are straightforward and simple, they may not represent haplotype blocks in a true biological sense. More sophisticated approaches may model the true haplotype blocks better. Commonly, LD based measures like D´ or *r^2^* are used for construction of haplotype blocks (Devlin and Risch, 1995). Furthermore, prior information of interaction between adjacent markers may help model local epistasis (Liu et al., 2019), however, difficulties in computing higher order interactions limits the size of haplotype blocks of that type. Haplotype blocks are assumed to be in higher linkage disequilibrium with QTL, and it was proven that haplotype blocks are able to capture local epistasis of markers in close proximity (Jiang et al., 2018). Furthermore, it has been suggested that the problem of apparent or phantom epistasis, which occurs between markers and QTL in incomplete LD, can be overcome with haplotype blocks (Wood et al., 2014; de los Campos et al., 2019). Hence it can be assumed, that haplotype blocks may improve genomic prediction.

In genomic selection, there is evidence that markers grouped to haplotype blocks can improve genomic prediction (Cuyabano et al., 2014; Jiang et al., 2018; Ballesta et al., 2019), while other studies delivered evidence against improving predictions (Solberg et al., 2008). Even with the methods described above for construction of haplotype blocks, it is always necessary to set appropriate hyperparameters like window size or an LD threshold to define block boundaries. Most previous studies in this area investigated a small range of LD thresholds, adjacent markers or window sizes in association studies and genomic prediction (Cuyabano et al., 2014; Hess et al., 2017; Maldonado et al., 2019). However, in terms of genomic prediction for plant breeding the huge variety of options and hyperparameters possible to construct haplotype blocks were not assessed in detail. Hence, the present study sought to investigate the following questions: 1.) How does the method of building haplotype blocks and its parameters affect the number of haplotypes? 2.) Are haplotype block predictions different from SNP predictions in terms of prediction accuracy? 3.) Is there a preferable haplotype construction method to improve genomic prediction?

These questions were addressed by employing various methods for constructing haplotypes, which are commonly discussed in the literature. The methods range from simple approaches such as marker adjacency (Villumsen and Janss, 2009; Villumsen et al., 2009; Jiang et al., 2018; Liang et al., 2020) and physical distances (Hess et al., 2017; Liang et al., 2020) to more sophisticated methods based on LD thresholds (Cuyabano et al., 2014; Voss-Fels et al., 2019; Bayer et al., 2021; Li et al., 2022) the confidence intervals of *D´* method described by Gabriel et al. (2002), the *Four-gamete Rule* method described by Wang et al. (2002) the *Solid Spine of LD* method (Barrett et al., 2005) and *“HaploBlocker”* (Pook et al., 2019), using four example datasets from canola, maize, wheat and soybean. To assess prediction accuracy, genomic prediction was performed using GBLUP, Bayesian LASSO, EGBLUP and RKHS models.

## 2 Materials and methods

### 2.1 Datasets

The datasets examined in this study are all publicly available. The canola dataset is from a spring-type canola hybrid breeding program (Jan et al., 2016). Briefly, 475 double haploid (DH) pollinators were crossed with two male sterile lines to create 950 F_1_ test hybrids. The hybrids where subsequently tested for seed yield, flowering time, field emergence, lodging, oil yield and glucosinolate content. For 910 test hybrids the complete phenotypic records were available, and all parental lines were genotyped with the Illumina *Brassica* 60k SNP array (Clarke et al., 2016). The maize dataset is derived from 847 test hybrids from a diverse dent nested association mapping population described by Bauer et al. (2013) consisting of 10 half-sib DH families. Double haploid lines were all crossed to the common flint line UH007 and F1 hybrids were phenotypically analyzed for dry matter yield (DMY), dry matter content (DMC), plant height (PH), days till tasseling (DtTAS) and days till silking (DtSILK), as described by Lehermeier et al. (2014). All DH lines were genotyped with the Illumina MaizeSNP50 SNP array (Clarke et al., 2016). The wheat dataset, described in Voss-Fels et al. (2019), consists of 191 released wheat varieties from 1966 to 2013 that were tested under three agrichemical treatments for a wide range of agronomic traits including yield, biomass yield, falling number, days till heading, plant height, harvest index kernel spike^-1^, nitrogen use efficiency (NUE), powdery mildew resistance, protein content, protein yield sedimentation value spike m^-2^, stripe rust and thousand kernel weight (TKW). All lines were genotyped with the Illumina 15k wheat SNP array described in Soleimani et al. (2020). The soybean dataset consisted out of 1000 lines from the USDA Soybean Germplasm Collection (Grant et al., 2010) with phenotypic records for protein and oil content (PC, OC) (Bandillo et al., 2015). For all lines, genotypic information from the Illumina Infinium SoySNP50K BeadChip (Song et al., 2013) was available.

With the exception of the maize dataset, all phenotypic data represented adjusted trait means per genotype. The published field data from the maize population was adjusted following methods used for phenotypic data analyses from the original publication.

### 2.2 Genotypic data

With exception of the canola dataset, physical SNP marker positions were obtained from the respective reference genome assemblies used in the original publications, namely the *Brassica napus* Express 617 genome (Lee et al., 2020), the maize B73 AGPv2 genome (Schnable et al., 2009), the wheat Chinese Spring IWGCS reference Sequence v1.0 (Zimin et al., 2017) and the soybean Glyma1.01 reference (Schmutz et al., 2010). In general, only markers with a unique physical position on the reference genome, a minor allele frequency ≥ 0.05 and a maximum of 10% missing values in each population were used for further analyses. This left a total of 29385, 32363, 8710 and 35821 markers for the canola, maize, wheat and soybean datasets, respectively. This corresponds to a marker density of 31.78, 15.63, 0.57 and 37.48 SNPs mbp^-1^ in canola, maize, wheat and soy respectively. After filtering, markers were imputed with the software “*BEAGLE”* V5.2 (Browning and Browning, 2007; Browning et al., 2018).

### 2.3 Haplotype block construction

We considered seven haplotype block construction methods based on (i) pre-determined LD thresholds, (ii) fixed windows of adjacent markers, (iii) fixed windows of adjacent base pairs, (iv) *“HaploBlocker”* (Pook et al., 2019), (v) the confidence intervals of *D´* method described by Gabriel et al. (2002), (vi) the *Four-gamete Rule* method described by Wang et al. (2002) and (vii) the *Solid Spine of LD* method (Barrett et al., 2005). The first three methods were implemented in the r package *“SelectionTools”* (downloadable at http://population-genetics.uni-giessen.de/~software/), while the latter three are implemented in the software “*Haploview”* v4.1 (Barrett et al., 2005). The different approaches are described in detail below. These methods were selected for their widespread use in haplotype block formation and their distinct characteristics. Methods such as the pre-determined LD threshold, confidence intervals of *D’*, the *Four-gamete Rule*, and the *Solid Spine of LD* are based on linkage disequilibrium (LD) and gamete frequency. They aim to model historical recombination hotspots and generate meaningful blocks within populations. However, these blocks do not necessarily represent functional groups. Therefore, we also included methods based on fixed windows to assess blocks that would not be constructed based on population-based measures alone. Additionally, while most methods consider block borders across the entire population, it is important to note that subpopulations or genotypes may have different recombination patterns. To account for this, we utilized the method *“HaploBlocker”* described in Pook et al. (2019) to construct haplotype blocks specific to different groups.

#### 2.3.1 LD threshold

LD between markers on the same chromosome was calculated as *r^2^* (Hill and Robertson, 1968) in *“SelectionTools”*. Haplotype blocks were built by starting with the two neighboring markers with the highest LD. If the pairwise LD exceeded a certain threshold, those markers were then assigned to a haplotype block. In the next step, if the LD between the next immediately adjacent markers and the markers at the block border again exceeded the threshold, the block was extended. This was done until no more markers fulfilled this criterion and the algorithm started over again with new markers. To account for misplaced markers, a tolerance parameter of 1 was used, meaning that one marker that did not fulfill the LD threshold was accepted if the next flanking marker fulfilled the LD criterion. Thresholds were set sequentially from 0.01 to 1 with a step size of 0.01, resulting in 100 different LD thresholds. Using very high thresholds to form blocks effectively eliminates redundant information, making these scenarios similar to LD pruning, which has been shown to improve prediction accuracy (Ye et al., 2019). On the other hand, very low thresholds result in the formation of large blocks commonly observed in introgression breeding, where recombination is sometimes very limited (Hao et al., 2020).

#### 2.3.2 Fixed windows of adjacent markers

Starting at the beginning of each chromosome, haplotype blocks consisting of *m* neighboring markers were constructed until all markers on a chromosome were assigned to blocks. We considered ⌈2*^x^* ⌉ markers with *x* being {1, 1.5, 2, 2.5 …}, until in the most excessive case all markers of a chromosome represented a haplotype block containing all markers of that chromosome. We chose to create blocks of such large size to address scenarios where entire chromosomes or large segments play an important role in traits, as well as scenarios related to introgression breeding, where recombination is limited (Hao et al., 2020).

#### 2.3.3 Fixed windows of adjacent base pairs

Starting at the beginning of each chromosome, haplotype blocks of *m* consecutive base pairs were constructed until the whole chromosome was partitioned into blocks. We considered ⌈2*^x^* ⌉ base pairs with *x* being {10, 10.5, 11, 11.5 …} until in the most excessive case a whole chromosome represented a block. Similar to the approach using fixed windows of adjacent markers, we selected to construct blocks of considerable size to accommodate scenarios where entire chromosomes or large segments influence traits, as well as situations related to introgression breeding characterized by limited recombination (Hao et al., 2020).

#### 2.3.4 HaploBlocker

Since different subpopulations might result in different block borders, we also built haplotype blocks with the algorithm of Pook et al. (2019). This algorithm relies on linkage instead of linkage disequilibrium to construct haplotype blocks. Here blocks are defined as consecutive sequence of genetic markers with a predefined frequency, a sequence of haplotype merging and splitting steps is applied to construct subgroup-specific haplotype blocks. This algorithm allows subgroup specific haplotype block borders. The algorithm was conducted with default settings with the r package *“HaploBlocker”* (Pook et al., 2019).

#### 2.3.5 Gabriel algorithm

The algorithm developed by Gabriel et al. (2002) (GAB) for the Human Haplotype Map generates 95% confidence bounds on *D´* between all intrachromosomal marker pairs. Marker pairs are considered in “strong LD” if the one-sided upper 95% *D´* confidence bound is higher than 0,98 and the lower bound is higher than 0.7. Markers in “strong LD” are consequently grouped into blocks. Blocks are extended until the outermost marker pairs don´t fulfill this criterion anymore.

#### 2.3.6 Four gamete rule

The *Four Gamete Rule* (GAM) described by Wang et al. (2002) groups consecutive markers into haplotype blocks if no evidence for a historical recombination event can be found between all marker pairs of a block. A historical recombination is defined if all four haplotypes of the new marker and any other previous marker are found with at least 1% frequency. If this is the case, a block border is created between those markers and the algorithm starts with a new block.

#### 2.3.7 Solid spine of LD

The *Solid Spine of LD* method (SPI), introduced by the developers of “*Haploview*” (Barrett et al., 2005), searches for a spine of strong LD by calculation of LD between all intrachromosomal marker pairs. In this method, two markers on the same chromosome form a block border if the pairwise *D´* is higher than 0.8. All markers in that window form the block. This allows for intermediate markers to not be in LD.

### 2.4 Genomic prediction models

In total, four genomic selection models were used to predict testcross (maize, canola) and inbred line (soybean, wheat) performance, respectively. The models represent two variations of the GBLUP and two models implemented in a Bayesian framework. The frequentist models were GBLUP (Bernardo, 1994; Meuwissen et al., 2001; VanRaden, 2008) and extended GBLUP to account for second-order additive*additive epistasis, following the EGBLUP model of Jiang and Reif (2015). The Bayesian model included the Bayesian LASSO model (Park and Casella, 2008) which offers the capability of marker-specific shrinkage, and the semiparametric RKHS regression model (de los Campos et al., 2009) which allows modeling of higher order epistasis.

In the GBLUP and EGBLUP the underlying model is assumed to be:


$$y=X\beta +Z_{a}a+Z_{i}i+e$$

where y is a vector of observations for a trait under consideration,  β is a vector of fixed non-genetic effects, a is a vector of random additive effects, i is a vector of random epistatic effects and e is the random residual term. $Z_{a}$ and $Z_{i}$ are design matrices relating the random effects to the phenotypic records. X is the design matrix for fixed effects and, in the case of the canola and soybean datasets, a vector of ones modeling the intercept ( $1_{n}\mu$ ). In the wheat dataset, two additional fixed effects for N fertilization and fungicide treatment were added, while in the maize dataset an additional 10 columns were added to assign individuals to half-sib families.

It is assumed that


$$a\sim N\left(0,G\sigma_{a}^{2}\right),\;i\sim N\left(0,G_{aa}\sigma_{aa}^{2}\right)\;and\;e\sim N\left(0,I\sigma_{e}^{2}\right)$$

where $\sigma_{a}^{2},\sigma_{aa}^{2}$ and $\sigma_{e}^{2}$ are additive genetic variance, epistatic genetic variance and residual variance respectively. G and $G_{aa}$ are the additive and epistatic relationship matrices, respectively. I is an identity matrix. Depending on inclusion of epistatic effects the epistasis terms were included or omitted.

The additive genomic relationship matrix was calculated following VanRaden (2008):


$$G=\frac{ZZ´}{2\sum^{}p_{i}\left(1-p_{i}\right)}$$

with the elements of Z being (0-2p_i_) for genotype H_i_H_i_, (1-2p_i_) for genotype H_i_H_j_ and (2-2p_i_) for genotype H_j_H_j_, where H_j_ is the haplotype (treated as a single marker) within a haplotype block, H_i_ is any other haplotype within that haplotype block except H_i_, and $p_{i}$ is the frequency of the *i*th haplotype in a haplotype block. Haplotype blocks with only two haplotypes were treated like standard biallelic markers. For the canola dataset, prior to construction of the genomic relationship matrix, parental genotypes were crossed *in silico* to derive hybrid genotypes, as described by Werner et al. (2018a).

According to Henderson (1985) and Jiang and Reif (2015), the second order (additive*additive) epistatic relationship matrix can be approximated with $G_{aa}=G\#G$ , with # denoting the pointwise (hadamard) product operation.

GBLUP and EGBLUP were implemented and solved with the R package *sommer* (Covarrubias-Pazaran, 2016; Covarrubias-Pazaran, 2018).

The general formula describing the model Bayesian LASSO model of Park and Casella (2008) is:


y=Xβ+Mf+e

where y is the vector of observations for a trait under consideration, β is a vector of fixed non-genetic effects, a is a vector of additive effects. X is the design matrix as described in the GBLUP section. M is an incidence matrix relating phenotypic records with the respective marker/haplotype profiles coded 0, 1, 2. The coefficients of the fixed ( β ) effects are assigned flat priors, while the coefficients of the marker/haplotype effects ( f ) are assigned double-exponential priors. This allows the shrinkage of some marker/haplotype effects to effectively zero, introducing sparsity into the model. This model was tested because we assumed that some marker variants and particularly some haplotypes would have no effect on some traits. Here, e is the random residual term. In the Bayesian LASSO, only additive effects were modeled, because additional effects in this framework would increase the computational burden to an unacceptable degree. This model was conducted in the r software with the package *BGLR* (Pérez and de los Campos, 2014) using the default parameters.

Following de los Campos et al. (2009) with kernel averaging, the RKHS model has following form:


$$y=X\beta +\sum_{l=3}^{L}u_{l}+e$$

with


$$p\left(\beta ,u_{1},\ldots u_{L},e\right)\propto \;\prod_{l=1}^{L}N\left(u|0,\;K_{l}\sigma_{ul}^{2}\right)\;N(e|0,\;I\sigma_{e}^{2})\;$$

where $K_{l}$ is an n*n kernel. It is calculated from the Euclidean distance between genotypes based on their marker/haplotype profile. We selected a Gaussian kernel with the *l*th value of the bandwidth parameter {0.1, 0.5, 2.5}. Xβ is treated in a similar manner to the Bayesian LASSO and $u_{l}$ is assumed to be the random genomic effect. That way the different random effects, i.e. the three kernel matrices from the three bandwidth parameters, are weighted by their variance components. Here, e is the random residual term. As for the Bayesian LASSO, the RKHS model was conducted in the r software with the package *BGLR* (Pérez and de los Campos, 2014) using the default parameters.

### 2.5 Genomic relationship

Generally, constructing haplotype blocks applies a transformation to the original marker data. To assess how well the marker data is also captured by haplotype blocks, we used the relationship coefficients obtained from the relationship matrix calculated following VanRaden (2008) (see above) and calculated the Pearson correlation between relationship coefficients obtained from SNPs and those obtained from haplotype blocks.

### 2.6 Evaluation of prediction accuracy

For all the four datasets, model performance was assessed by running 100 cross-validation runs, where each cycle consisted of splitting the population into 80% training population and 20% validation population. Each model was trained on the training population and then this model was used to predict the validation population with masked phenotypic data. Furthermore, in the maize dataset, a family wise cross validation was conducted. This was done to test how predictive haplotype blocks are to predict genetically distant individuals. Here, the dataset was split according to the family assignment of the nested association mapping population and each family served once as validation set. In both cross validation schemes, the Pearson correlation coefficient (r) between observed and predicted phenotypic values of the validation population was used as a measure of prediction accuracy.

## 3 Results

### 3.1 Haplotype block properties

In all the datasets analyzed, haplotypes of varying sizes were examined. The haplotype blocks had average physical sizes ranging from 1.02 kbp to 47453.13 kbp, 379625.06 kbp, 1073741.82 kbp, and 47453.13 kbp, respectively, for canola, maize, wheat and soybean. A summary of the average size distributions can be found in **Table 1**. Notably, the fixed window approaches allowed for the construction of both the smallest haplotype blocks (1.02 kbp) and the largest haplotype blocks (**Table 1**).

**Table 1:** Average size ranges of haplotype blocks constructed by LD, fixed window of adjacent markers and fixed window of adjacent base pairs in the canola, maize, wheat and soybean dataset.

Dataset	Method	minimal average size (kbp)	maximal average size (kbp)
Canola	LD	97.49 (*r^2^ = *1)	2629.87 (*r^2^ = *0.01)
	fixed window of adjacent marker	25.46 (*nSNP* = 2)	39801.09 (*nSNP* = 2048)
	fixed window of adjacent base pairs	1.02 kbp	47453.13 kbp
Maize	LD	8.08 (*r^2^ = *1)	21556.53 (*r^2^ = *0.01)
	fixed window of adjacent marker	64.31 (*nSNP* = 2)	205312.88 (*nSNP* = 5793)
	fixed window of adjacent base pairs	1.02 kbp	379625.06 kbp
Wheat	LD	106.79 (*r^2^ = *1)	64954.10 (*r^2^ = *0.01)
	fixed window of adjacent marker	1544.58 (*nSNP* = 2)	667692.8 (*nSNP* = 1024)
	fixed window of adjacent base pairs	1.02 kbp	1073741.82 kbp
Soybean	LD	138.55 bp (*r^2^ = *1)	1587.07 (*r^2^ = *0.01)
	fixed window of adjacent marker	430.27 (*nSNP* = 2)	1526.61 (*nSNP* = 2897)
	fixed window of adjacent base pairs	1.02 kbp	47453.13 kbp

**Figure 1:**
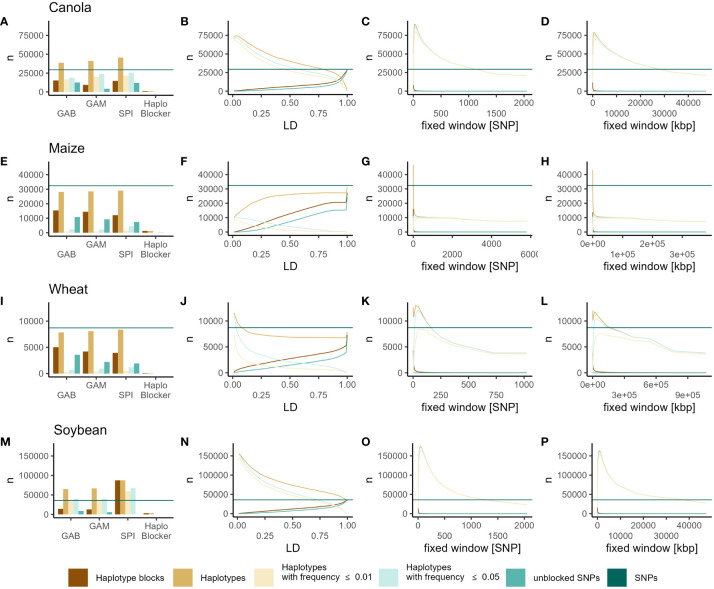
Numbers of SNPs (dark green horizontal line), haplotype blocks (dark orange), haplotypes (light orange), haplotypes with frequency ≤ 0.05 (light green), haplotypes with frequency ≤ 0.01 (light yellow) and unblocked SNP markers (green) identified by GAB, GAM, SPI, *“HaploBlocker”* **(A, E, I, M)**, LD **(B, F, J, N)**, fixed window of adjacent markers **(C, G, K, O)** and fixed window of adjacent base pairs **(D, H, I, P)** in canola **(A–D)**, maize **(E–H)**, wheat **(I–L)** and soybean **(M–P)**.

Within all datasets using the methods implemented in *“Haploview”* (GAB, GAM, SPI), the number of haplotype blocks was consistently lower than the number of total SNPs (**Figures 1A, E, I, M**). However, a significant portion of those blocks consisted of only a single SNP (unblocked SNPs) (**Figures 1A, E, I, M**). Moreover, the total number of haplotypes available for genomic prediction (excluding single SNP blocks) increased in the canola and soybean datasets, remained similar to the number of SNPs in wheat, and decreased in maize (**Figures 1A, E, I, M**). Across all datasets, the number of blocks based on LD increased with higher LD thresholds. Additionally, in the case of maize, the number of haplotypes exhibited a similar pattern. With LD-based haplotype blocks, the number of haplotypes (excluding single SNP blocks) exceeded the total number of SNPs across all LD thresholds in soybean and was lower across all thresholds in maize (**Figures 1B, F, J, N**). In canola, thresholds above *r^2^ = *0.75 resulted in fewer haplotypes than SNPs, while lower thresholds yielded higher numbers. Conversely, in wheat, only relatively small blocks (*r^2^* ≤ 0.10) increased the number of haplotypes compared to the number of SNPs (**Figure 1B**). With fixed window blocks, the number of haplotype blocks generally decreased with increasing block size (**Figures 1C, D, G, H, K, L, O, P**). Here, the number of haplotypes was the highest with relatively small blocks, with increasing block size, the number of haplotypes decreased (**Figures 1C, D, G, H, K, L, O, P**). Notably, in comparison to SNPs, the number of haplotypes was higher for blocks smaller than 1024, 6, 128, and 1449 SNPs, or 23726.57 kbp, 92.68 kbp, 134217.73 kbp, and 33554.43 kbp in the canola, maize, wheat, and soybean datasets, respectively (**Figures 1C, D, G, H, K, L, O, P**). In all scenarios, increasing block size resulted in fewer unblocked markers, especially with the fixed window approaches. In all datasets, the *“HaploBlocker”* method produced the fewest haplotypes, considerably fewer than the number of SNPs (**Figures 1A, E, I, M**). Furthermore, across all datasets and methods, except for blocks based on *“HaploBlocker”*, most of the introduced haplotypes can be classified as rare (Frequency ≤ 0.05) or very rare (Frequency ≤ 0.01) (**Figure 1**).

Across the four datasets, the examination of the correlations between relationship coefficients derived from SNPs and haplotypes revealed high redundancy between the two marker types in many method/parameter combinations. The methods implemented in *“Haploview”* resulted in relationship coefficients that were highly correlated to those obtained from SNPs, closely approaching a correlation coefficient of 1, in canola, wheat, and soybean (**Figures S1A, E, I, M**). However, in maize, these methods only produced intermediate correlations (GAB = 0.60, GAM = 0.50, SPI = 0.46) (**Figure S1E**). In all datasets, relationship coefficients from haplotypes from LD-based haplotype blocks were highly correlated to those obtained from SNPs (*r* > 0.75) with little variation observed across LD thresholds. Only at very low LD thresholds, this correlation was slightly lower, while it was slightly higher for very high thresholds (**Figures S1B, F, J, N**). Additionally, small fixed window blocks resulted in relationship coefficients similar to those obtained from SNPs, closely approaching a correlation coefficient of 1. However, this similarity eroded drastically with increasing block size (**Figures S1C, D, G, H, K, L, O, P**). Notably, in Soybean, while the correlation between relationship coefficients from SNPs and haplotypes decreased with increasing block size of the fixed window of adjacent base pairs, it slightly increased again with the largest blocks (*nKB* = 67108.86) (**Figure S1P**). In canola and soybean, relationship coefficients obtained from *“HaploBlocker”* were highly correlated to those obtained from SNPs (**Figures S1A, M**). In wheat, this correlation was lower (r = 0.75), and in maize, it was close to zero (r = 0.058), indicating that these blocks capture different information (**Figures S1E, I**).

### 3.2 Genomic prediction

#### 3.2.1 Canola

**Figure 2:**
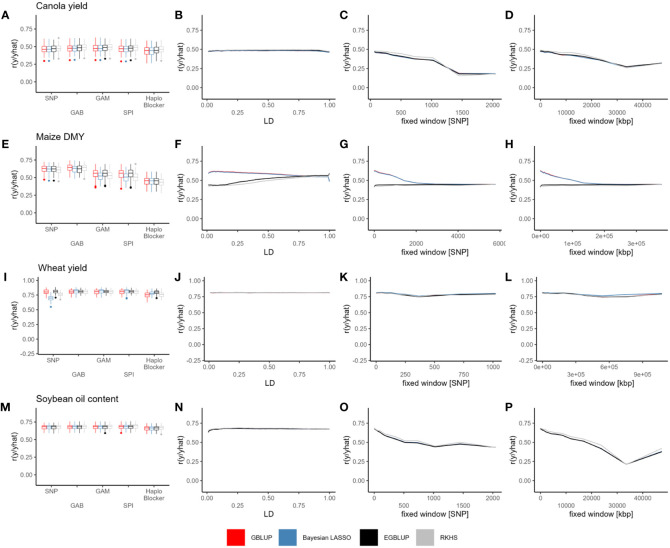
Prediction accuracy (r) of GBLUP (red), Bayesian LASSO (blue), EGBLUP (black) and RKHS (grey) with SNPs, GAB, GAM, SPI, *“HaploBlocker”* **(A, E, I, M)**, LD **(B, F, J, N)**, fixed window of adjacent markers **(C, G, K, O)** and fixed window of adjacent base pairs **(D, H, L, P)** based haplotype blocks, in canola seed yield **(A–D)**, maize DMY **(E–H)**, wheat seed yield **(I–L)** and soybean oil content **(M–P)**. Individual points in the line plots represent the mean over all cross validation runs for each haplotype block parameter and model combination.

Within the canola dataset, the prediction accuracy across different models ranged from 0.3 to 0.85, with a strong dependence on the specific trait. Notably, for oil yield, field emergence, glucosinolate content, and lodging, the models considering epistatic effects (EGBLUP and RKHS) consistently outperformed by the other SNP-based models (**Figure S2**). However, this effect did not consistently translate to haplotype-based predictions. Prediction accuracy showed little variation across LD threshold as well as between LD base, *“Haploview”* or *“HaploBlocker”* methods (**Figures 2A, B**, **S2**). On the other hand, the fixed-window approaches exhibited the most variation, with a substantial decrease in prediction accuracy as the block size increased for every trait, while small blocks based on fixed windows resulted in prediction accuracies similar to those based on SNPs or the remaining methods (**Figures 2C, D**, **S2**).

Comparing haplotype blocks to SNP-based prediction, the improvement in prediction accuracy ranged from 0.007 to 0.021 for GBLUP, 0.008 to 0.024 for Bayesian LASSO, 0.008 to 0.023 for EGBLUP, and 0.007 to 0.022 for RKHS. These values were based on the haplotyping method that yielded the highest prediction accuracy for each specific trait and model (**Tables 2**, **S1**). Interestingly, the use of haplotypes seemed to have the least impact on oil yield (**Figure S1**; **Table S1**). Except for flowering time with RKHS, the LD-based methods generally resulted in the most significant improvements. However, no ideal LD threshold or range of thresholds could be identified (**Table S1**). In the case of flowering time with RKHS, the optimal haplotyping method involved a fixed window of adjacent base pairs measuring 20987.15 kbp.

**Table 2:** Average prediction accuracy of SNP based prediction compared to the best haplotyping method of canola, maize, wheat and soybean for some example traits.

Dataset	Trait	Model	SNP prediction accuracy	Best haplotyping algorithm	Prediction accuracy by best haplotyping algorithm	Improvement by best haplotyping algorithm
Canola	yield	GBLUP	0.464	*r^2^ = *0.6	0.485	0.021
		Bayesian LASSO	0.462	*r^2^ = *0.59	0.486	0.024
		EGBLUP	0.471	*r^2^ = *0.6	0.492	0.021
		RKHS	0.474	*r^2^ = *0.71	0.496	0.022
	flowering time	GBLUP	0.709	*r^2^ = *0.12	0.721	0.012
		Bayesian LASSO	0.697	*r^2^ = *0.15	0.721	0.024
		EGBLUP	0.711	*r^2^ = *0.14	0.723	0.012
		RKHS	0.704	*nKB* = 2097.15	0.719	0.015
Maize	DMY	GBLUP	0.624	GAB	0.635	0.011
		Bayesian LASSO	0.616	GAB	0.621	0.006
		EGBLUP	0.620	*nSNP* = 8	0.622	0.002
		RKHS	0.608	GAB	0.631	0.023
	DtTAS	GBLUP	0.847	*nSNP* = 4	0.847	0.000
		Bayesian LASSO	0.846	*r^2^ = *1	0.842	-0.004
		EGBLUP	0.845	*nSNP* = 4	0.846	0.000
		RKHS	0.846	*r^2^ = *1	0.842	-0.003
Wheat	yield	GBLUP	0.805	*r^2^ = *0.23	0.813	0.008
		Bayesian LASSO	0.697	*nSNP* = 46	0.818	0.122
		EGBLUP	0.811	*r^2^ = *0.1	0.815	0.005
		RKHS	0.765	*r^2^ = *0.1	0.814	0.049
	sedimentation value	GBLUP	0.493	*nKB* = 1073741.82	0.619	0.126
		Bayesian LASSO	0.488	*nKB* = 1073741.82	0.636	0.148
		EGBLUP	0.620	*nKB* = 1073741.82	0.627	0.006
		RKHS	0.610	*nKB* = 1073741.82	0.631	0.021
Soybean	oil content	GBLUP	0.674	*r^2^ = *0.24	0.682	0.008
		Bayesian LASSO	0.675	*r^2^ = *0.24	0.683	0.008
		EGBLUP	0.674	*r^2^ = *0.24	0.682	0.008
		RKHS	0.677	*r^2^ = *0.26	0.691	0.014
	protein content	GBLUP	0.601	*nSNP* = 4	0.606	0.006
		Bayesian LASSO	0.602	*nSNP* = 4	0.608	0.006
		EGBLUP	0.609	*nSNP* = 4	0.611	0.003
		RKHS	0.609	*nSNP* = 4	0.613	0.003

#### 3.2.2 Maize

Prediction accuracy obtained from the random cross validation ranged from 0.4 to 0.9 and was trait-dependent. Here, little difference between models was observed with SNP-based prediction (**Figures 2E**, **S3**). With haplotypes, however, there were considerable differences between Models implemented in a Bayesian framework and frequentist models (**Figures 2**, **S3**). With haplotypes based on LD, prediction accuracy decreased with higher LD thresholds for GBLUP and EGBLUP and increased for Bayesian LASSO and RKHS, respectively (**Figures 2F**, **S4**). Here for DMY, DMC and PH, respectively, all models approached a similar prediction accuracy around *r^2^* ~0.75 (**Figure S3**). And for DtTAS and DtSILK all models approached the same prediction accuracy around *r^2^* ~0.55 (**Figure S3**). The same behavior could not be observed with the fixed window haplotypes, where prediction accuracy obtained from GBLUP and EGBLUP decreased drastically with increasing block size. Here, for models implemented in a Bayesian framework the prediction accuracy remained low independent of the block size (**Figures 2G, H**, **S3**). Except for DMY, where the GAB method slightly improved prediction accuracy, haplotypes based on the algorithms implemented in “*Haploview”* decreased prediction accuracy in every scenario (**Figures 2E**, **S4**). In general, there was no discernable improvement of prediction accuracy by haplotypes compared to SNP-based predictions. In all traits but DMY, the haplotyping method with the highest prediction accuracy even decreased prediction accuracy with Bayesian LASSO and RKHS (**Tables 2**, **S1**), whereas for GBLUP and EGBLUP prediction accuracy did not or only slightly increased prediction accuracy compared to SNP based prediction. DMY profited most from haplotypes, whereby GBLUP, Bayesian LASSO and RKHS worked best with the GAB method while for EGBLUP a fixed window of 8 SNPs was ideal (**Figure 2**; **Table 2**). Besides DMY, for Bayesian LASSO and RKHS haplotypes worked best with an LD threshold of *r^2^ = *1, however, prediction accuracy was still worse than SNP based prediction (**Table S1**; **Figure S4**). For the same traits, Frequentists model worked best with varying fixed window size haplotypes, with a maximal improvement of 0.002 (**Table S1**). The *“HaploBlocker”* method together with very large fixed window blocks yielded the lowest prediction accuracies across all traits.

The family-wise cross validation generally yielded considerably lower prediction accuracies than its random counterpart (**Figure S4**; **Table S2**). The ranking in prediction accuracies obtained from haplotype blocks followed the pattern of the random counterpart, albeit being lower (**Figure S4**; **Table S2**). Mentionable, prediction accuracy approached zero for the *HaploBlocker”* method together with very large fixed window blocks.

#### 3.2.3 Wheat

Prediction accuracy in the wheat dataset exhibited much greater variability between traits compared to the other three datasets, ranging from -0.4 to 0.9, depending on the specific trait. Interestingly, even with SNP-based predictions, considerable differences in prediction accuracy were observed across (i) models that consider epistasis and those that do not, (ii) frequentist and models implemented in a Bayesian framework, and (iii) combinations of (i) and (ii) (**Figures S5**–**S7**). However, when haplotype blocks were utilized, all models achieved at least the average prediction accuracy of the best SNP-based model for 13 out of 15 traits (**Figures S5**–**S7**; **Table S1**). This was achieved by using haplotype blocks constructed with varying methods, including even the largest possible haplotype blocks based on fixed windows (e.g., using whole chromosomes as blocks) (**Figures 2**, **S5**–**S7**; **Table S1**).

Furthermore, for traits such as yield, biomass yield, NUE, protein yield, sedimentation value, stripe rust, and falling number, the previously worst-performing SNP-based model became the best-performing model when using haplotype blocks (**Table S1**). Additionally, for traits with very low or even negative prediction accuracy based on SNPs (e.g., plant height, TKW, days till heading, falling number, powdery mildew, and stripe rust), strong improvements were achieved through the use of haplotypes (**Figures S5**–**S7**; **Table S1**). Models implemented in a Bayesian framework seemed to benefit the most from the utilization of haplotypes, with changes in prediction accuracy ranging from -0.039 to 0.170 for GBLUP, from 0.006 to 0.277 for Bayesian LASSO, from -0.003 to 0.085 for EGBLUP, and from 0.025 to 0.291 for RKHS (**Tables 2**, **S1**). The most notable improvements were typically seen when prediction accuracy varied considerably between models using SNP data. Only for cases, such as falling number with RKHS, kernel spike^-1^ with EGBLUP, spike m^-2^ with RKHS and EGBLUP, and stripe rust resistance with GBLUP, did the prediction accuracy decrease compared to SNP-based prediction when using haplotype blocks (**Table S1**).

#### 3.2.4 Soybean

The prediction accuracy in the soybean dataset ranged from 0.5 to 0.8 and exhibited a striking similarity between oil content and protein content. No noticeable differences were observed between models based on SNPs (**Figures 2M**, **S8A, E**). Moreover, there was minimal variation in prediction accuracy across different LD thresholds, with only a slight decrease in accuracy observed between *r^2^ = *0.01 and 0.05 (**Figures 2N**, **S8B, F**).

When using fixed windows of adjacent marker blocks, the prediction accuracy experienced a decline with increasing block size for all models (**Figures 2O**, **S8C, G**). Similar behavior was observed for fixed windows of adjacent base pairs blocks, except for a marginal increase in prediction accuracy with blocks of size 47453.13 kbp (**Figures 2P**, **S8D, H**). However, it is worth noting that the prediction accuracy remained lower than the SNP-based prediction in that case. Overall, the improvements achieved with haplotypes were relatively minor (**Tables 2**, **S1**). For oil and protein content, the best haplotype block method and parameter improved the prediction accuracy by only 0.006 and 0.008 with GBLUP, 0.006 and 0.009 with Bayesian LASSO, 0.003 and 0.008 with EGBLUP, and 0.003 and 0.014 with RKHS, respectively, compared to the SNP-based prediction (**Tables 2**, **S1**).

Interestingly, within the traits, it was observed that the models worked best with the same haplotype block method: an LD threshold of *r^2^ = *0.24-0.26 for oil content and a fixed window size of *nSNP* = 4 for protein content.

## 4 Discussion

Using datasets from four diverse crops and haplotype blocks constructed using a broad range of construction parameters, we show how haplotype blocks change in size and influence the effective number of predictors for genomic prediction. While haplotype blocks sometimes drastically change the number of predictors, genomic prediction accuracy was only marginally affected with no consistent improvement for any method and trait.

Haplotype blocks were built based on LD (*r^2^*), fixed window sizes of adjacent marker or base pairs as well as the three algorithms implemented in the software “*Haploview”* and the method *“Haploblocker”*. The *r^2^* measurement of LD between markers (Hill and Robertson, 1968; Hill, 1981) is highly correlated to *D´* (VanLiere and Rosenberg, 2008), which is more commonly used in tagSNP methods where it showed superior performance to other measures (Carlson et al., 2004; de Bakker et al., 2005). According to Cuyabano et al. (2014), *r^2^* and *D´* show no difference in terms of prediction accuracy in genomic prediction. The high resolution of haplotype blocking methods and construction parameters allowed an examination of a wide range of haplotype block sizes that are normally not considered in genomic prediction. Most studies in this regard only include single or few construction methods or parameters (Lorenz et al., 2010; Ballesta et al., 2019; Maldonado et al., 2019), although our results show that the method of haplotype construction can potentially impact prediction quality. We included haplotype blocks of relatively large sizes, such as a LD threshold of 0.01 and whole chromosome blocks, which may initially seem unrealistic. However, we included these large blocks to account for scenarios in which traits are controlled by large chromosome segments (Voss-Fels et al., 2019), possibly resulting from introgression breeding with suppressed recombination (Hao et al., 2020).

Here, in three datasets the number of haplotypes could be increased substantially compared to the number of SNPs. The number of haplotypes we observed in the four examined datasets was lower than observed in cattle (Cuyabano et al., 2014; Cuyabano et al., 2015; Li et al., 2021; Li et al., 2022) and human (Liang et al., 2020) but similar to previous reports in plants including *Eucalyptus globulus* (Ballesta et al., 2019), maize (Matias et al., 2017) and rice (Matias et al., 2017). These variations may arise from differences in population diversity, marker density, and sequencing technology. The haplotype number detected in maize by Matias et al. (2017) was comparable to that observed in our analysis using around ten times fewer SNP markers, indicating that haplotype number is not (solely) dependent on marker density. However, as expected there is a relationship between the population size and haplotype number, with more (diverse) genotypes causing more haplotypes. The number of haplotypes we detected corresponded to the population size used for each crop, with wheat having the fewest haplotypes and soybean the most, independent of the method. Nevertheless, an effect of genetic diversity within a species or population cannot be discounted without comparative within-species analyses of alternative populations. Some authors argue that use of haplotype blocks can help to reduce dimensionality (Kim et al., 2019; Pook et al., 2019). However, depending on the methods and parameters for haplotype construction the number of haplotypes was sometimes higher in the examined datasets than the number of SNPs. This may reflect lower marker numbers and different methods compared to Kim et al. (2019). Dimensionality can certainly be decreased if rare haplotypes would be excluded (Hess et al., 2017; Li et al., 2022). The method *“HaploBlocker”* described by Pook et al. (2019) decreased the dimensionality in every examined dataset. In all cases, the major drawback of the large number of additional variants is the very low frequency at which the haplotypes occur. However, low frequency variants are often assumed to be in higher LD with recent causal mutations (Bloom et al., 2019; Wainschtein et al., 2022), implying that their detection and use for predictions could be beneficial. However, caution is needed when considering all haplotypes, especially rare ones. In genomic predictions. effect estimation of rare variants require large populations to be estimated accurately (Meuwissen et al., 2001; Goddard and Hayes, 2007). In large populations, rare variants can be observed at higher frequencies which enables a more accurate estimation of their trait effects. In SNP based prediction markers are commonly excluded if they have a minor allele frequency ≤ 0.05 (Technow et al., 2012; Crossa et al., 2013; Jan et al., 2016; Werner et al., 2018a; Zhang et al., 2018). With large populations, filtering could be shifted from frequencies to allele counts, potentially leading to more reliable effect estimates of rare haplotypes. However, increasing the population could again increases the number of rare new haplotypes. In all four datasets, the number of unblocked SNPs decreased with increasing block size. With LD based haplotype blocks, increasing the LD threshold resulted in an increase of unblocked SNPs.

Genomic prediction was conducted using four models: GBLUP, EGBLUP, Bayesian LASSO, and RKHS regression, with the latter two implemented within a Bayesian framework. GBLUP, being the golds standard of genomic prediction, is a widely employed prediction models in breeding, hence we included it in the analysis. However, GBLUP assumes that all markers or haplotypes contribute to the trait (through relationship), prompting the inclusion of Bayesian LASSO, which allows for marker or haplotype-specific shrinkage of effects towards zero. This is beneficial in scenarios where not all markers or haplotypes have an impact on the trait. Given the assumption that haplotypes capture local epistatic effects (Jiang et al., 2018), EGBLUP and RKHS regression were employed to assess whether considering global epistasis between haplotype blocks could yield a substantial improvement in genomic prediction. Although haplotype blocks are typically fewer in number compared to SNPs, the number of haplotypes used for prediction was often comparable to or even greater than the number of SNPs. Therefore, we selected prediction models capable of handling the challenges posed by the large p small n scenario, opting not to explore machine learning models. Furthermore, the application of machine learning methods would have required extensive hyperparameter optimization, which would have significantly exceeded the computational time required for the four prediction models employed in this study. Lastly, the objective of this study was to compare various haplotype blocking methods and parameters, rather than comparing different prediction models.

Generally, genomic prediction accuracies based on SNPs were similar to those reported in the literature across all datasets. In the canola dataset, accuracies closely matched Jan et al. (2016), with a small improvement likely due to the higher number of markers remaining after filtering. Trait prediction accuracies in canola/rapeseed were mostly consistent with previous reports, with minor variations observed for field emergence, and glucosinolate content (Würschum et al., 2014; Jan et al., 2016; Werner et al., 2018a; Werner et al., 2018b). Also in the maize dataset, SNP-based genomic prediction accuracy roughly matched the original publication (Lehermeier et al., 2014), with expected differences due to varying cross-validation schemes. Maize hybrids exhibited high prediction accuracies as previously reported (Technow et al., 2012; Crossa et al., 2014; Millet et al., 2019). In wheat, prediction accuracies based on SNPs for seed yield and yield components were on a very high level (**Table S1**) compared to many previously published reports (Lado et al., 2013; Zhao et al., 2013; Crossa et al., 2014; Daetwyler et al., 2014; Crossa et al., 2016; Edwards et al., 2019). Furthermore, prediction accuracies based on SNPs for stripe rust resistance, despite population differences, showed a similar level than observed by Daetwyler et al. (2014). Whereas protein content had a higher prediction accuracy compared to Crossa et al. (2016), sedimentation value was predicted equally well. In soybean, prediction accuracies based solely on SNPs were comparable to levels reported by Jarquin et al. (2016) for oil content and protein content, despite considerable differences in the cross-validation and modeling schemes. The lack of differences in prediction accuracies may be explained by the narrow genetic diversity in soybean breeding material due to genetic bottlenecks (Hyten et al., 2006).

Genomic prediction with LD-based haplotype blocks in canola resulted in the highest accuracy improvements for most model/trait combinations. Variation in prediction accuracy across LD thresholds was minimal. The optimal threshold varied significantly by trait and model, ranging from very low (0.01) to high (0.89). In wheat, LD-based haplotype blocks were superior to the other haplotyping methods for 20 out of 60 model/trait combinations, but accuracy didn’t always improve compared to SNP-based prediction. Similar low variation across LD thresholds was observed in soybean. For soybean’s oil content, the ideal LD threshold for accuracy estimates across all models was 0.24-0.26. In maize, only the Bayesian LASSO and RKHS models achieved the highest improvements with LD based haplotype blocks with a threshold of 1, effectively removing redundant information. In this scenario, only markers in complete LD were grouped into a block, effectively removing redundant information. This process, is similar to LD pruning, which has been demonstrated to enhance prediction accuracy (Ye et al., 2019). Intriguing patterns were observed with LD-based haplotypes in maize, the two models implemented in a Bayesian framework (Bayesian LASSO and RKHS) behaved in an opposite direction to the other (frequentist) models, potentially due to different estimation procedures. In contrast to Cuyabano et al. (2014), we generally did not find an ideal LD threshold or even an ideal threshold specific to each dataset and mostly not even an ideal threshold within one trait. The prediction accuracy variation along LD thresholds reported in cattle (Cuyabano et al., 2014; Li et al., 2021; Li et al., 2022) was similar to the variation observed in our analyses. This suggests that any LD threshold is reasonable for genomic prediction due to low variation of prediction accuracy. We propose that even with extreme LD thresholds, reasonably accurate haplotype blocks are constructed, which explains the low variation observed across LD thresholds in all datasets. Additionally, in all datasets, the correlation between relationship coefficients obtained from markers and haplotypes was consistently high, with little variation across LD thresholds. This suggests that relationship representation remains consistent when using LD-based haplotype blocks.

The use of small fixed window blocks led to prediction accuracies comparable to those achieved with individual SNPs. Additionally, in maize, our findings aligned with those of Jiang et al. (2018) in Flint material, showing similar prediction accuracy patterns for frequentist models using small fixed window size haplotype blocks (2-5 markers). Interestingly, in maize, prediction accuracy eroded with the two frequentist models and increasing block size based on fixed windows, whereas for two Bayesian models the prediction accuracy was low across all parameters. Except for the wheat dataset, using excessively large fixed windows to build haplotype blocks considerably reduced prediction accuracy, as observed in previous studies with cattle (Hess et al., 2017). Unrealistically large blocks likely obscure the effects of true QTL within them. Furthermore, these larger blocks are generally more prone to errors in genotyping, and imputation, which accumulate in large blocks and limit prediction accuracy of genomic prediction models utilizing these blocks. These errors can also introduce false rare haplotypes, exacerbating issues related to rare variants. Additionally, as block size increases, haplotypes become more specific to genotypes or subpopulations, resulting in the absence of certain haplotypes in the training set but presence in the validation set. This lack of overlap leads to inaccurate estimation of the effects for those haplotypes, thus decreasing prediction accuracy due to the limited shared haplotypes between the training and validation sets. In the case of wheat, however, using very large blocks, such as whole chromosomes, resulted in considerable improvements in prediction accuracy. Mentionable improvements were observed for traits such as wheat stripe rust resistance, powdery mildew resistance, and kernel spike^-1^. This improvement can likely be attributed to introgression breeding in wheat, where large chromosome segments are introgressed and preserved due to restricted recombination (Hao et al., 2020). Furthermore, the wheat D-subgenome exhibits large LD haplotype blocks that are important for yield and biomass-related traits (Voss-Fels et al., 2019). However, it should be noted that these improvements were observed in cases where the model performance was initially at a very low level with SNPs. The correlation between relationship coefficients obtained from markers and haplotypes was high for small fixed window blocks but decreased as block size increased. This suggests that crucial relationship information is lost or encoded within large haplotype blocks, which cannot be accessed for accurate prediction. As a result, the prediction accuracy in canola, maize, and soybean is reduced. However, it is important to highlight that large blocks can potentially introduce additional trait information, as demonstrated by their impact in some of the wheat traits.

The widely used algorithms implemented in *“Haploview”* did not exhibit superiority in terms of prediction accuracy compared to other methods. Although the method proposed by Gabriel et al. (2002) showed a slight improvement, particularly in maize DMY, these gains remained modest when compared to SNP-based prediction. In contrast to the findings of Matias et al. (2017), our analysis generally revealed a decrease in prediction accuracy rather than a benefit from haplotypes based on *“Haploview”* in the maize dataset. This discrepancy could be attributed to differences in the plant materials studied. While Matias et al. (2017) examined a diverse collection of tropical maize lines, our analyses focused on European dent material characterized by a relatively strong population structure (Lehermeier et al., 2014). Moreover, the population studied by Matias et al. (2017) was nearly twice the size of our investigation, potentially leading to increased recombination events between loci and reducing the potential size of haplotype blocks. Another contributing factor may be the limited representation of relationship captured by those haplotypes, as evidenced by the intermediate correlation between relationship coefficients obtained from markers and haplotypes. In contrast, canola, wheat, and soybean exhibited a high correlation in this regard. Unlike the findings of Ma et al. (2016) suggest, our study did not observe improved prediction accuracies in soybean using the method proposed by Gabriel et al. (2002). This discrepancy could be attributed to several factors, including differences in the traits under examination, as well as substantial variations in population size and marker density. It is worth noting that the method proposed by Gabriel et al. (2002) shares similarities with the LD-based method described earlier, implying that haplotype blocks formed using this method may already be represented using a specific LD threshold.

The *“HaploBlocker”* method (Pook et al., 2020) has the advantage of constructing subgroup-specific haplotype blocks and was implemented to address this aspect. However, this approach did not improve prediction accuracy and even led to a decrease of prediction accuracy in some cases. In canola and soybean, haplotype blocks from *“HaploBlocker”* effectively captured the genomic relationship represented by SNPs. In wheat, the representation was reasonable, but in maize, it was notably inadequate. Similar to the large fixed windows, haplotypes generated by this method are specific to genotypes or subpopulations. Consequently, haplotypes present in the validation set may not be observed in the training set, resulting in the inability to estimate their effects accurately and leading to decreased prediction accuracy due to the limited number of shared haplotypes between the training and validation sets. Particularly in the maize population, which exhibited strong population structure, the *“HaploBlocker”* method resulted in comparatively low prediction accuracies. This was pronounced with the family-wise cross validation, where the accuracies were diminished to nearly zero. In this scenario, even when using SNPs, the number of shared alleles or haplotypes between the training and validation sets will be minimized. This effect will be particularly prominent when employing a method that constructs subgroup-specific blocks.

In general, with the exception of wheat, prediction accuracies based on haplotype blocks using GBLUP and EGBLUP followed the correlation observed between relationship coefficients obtained from SNPs and haplotype blocks. This suggests that a portion of the prediction accuracy achieved with haplotypes is derived from reinterpreting the SNP information. However, in the case of wheat, this pattern did not hold true, even when using large fixed window blocks. Furthermore, considerable prediction accuracy differences were observed across models for wheat traits, but these differences were consistently compensated for by utilizing haplotype blocks with varying methods and parameters. This indicates that additional information beyond genetic relatedness contributes to the prediction accuracy when using haplotype blocks. One possible explanation is that haplotype blocks are generally considered to exhibit higher LD with QTL compared to individual markers (Jiang et al., 2018).

Multiple factors contribute to the accuracy of genomic prediction. One crucial factor is the relationship among genotypes, which is overlooked in random cross-validation approaches. In such cases, closely related genotypes may be included in both the training and validation sets, leading to higher prediction accuracies for related individuals (Massman et al., 2013; Hickey et al., 2014; Werner et al., 2020). Consequently, the prediction accuracies obtained from random cross-validation are population-specific and cannot be readily adopted to all breeding populations (Werner et al., 2020). To address this issue, we conducted a family-wise cross-validation in the maize dataset to assess the predictive performance of haplotype blocks for less related individuals. As expected from Werner et al. (2020), we observed a decrease in prediction accuracy compared to random cross-validation. However, the relative ranking of haplotype block methods and parameters remained consistent with that of the random cross-validation, indicating no added benefit from haplotypes in predicting the breeding values of genetically distinct materials.

Moreover, GBLUP models trained with small haplotype blocks exhibited very similar prediction accuracies to models trained with SNPs. This is expected since haplotype effects can be partially defined as the sum of individual marker effects within their respective block. Another advantage of haplotype effects is their ability to capture local epistasis, as demonstrated by Jiang et al. (2018). However, it is worth noting that purely additive models, especially in prediction methods like GBLUP where marker effects are estimated simultaneously, already implicitly capture local epistasis among markers in complete LD.

The use of haplotypes has been proposed as a means to address the challenges associated with apparent or phantom epistasis (Wood et al., 2014). Apparent or phantom epistasis can occur when two markers are in incomplete LD with QTL, resulting in statistically significant marker interactions in association studies and enhanced prediction accuracies in genomic prediction with models considering epistasis (Wood et al., 2014; de los Campos et al., 2019; Schrauf et al., 2020). This effect may be particularly pronounced in the wheat dataset, which had a significantly lower marker density compared to the other three datasets. Consequently, the use of haplotype blocks sometimes led to considerable improvements in prediction accuracy.

There is a multitude of factors affecting the accurate assembly of haplotype blocks and their respective haplotypes. Especially in complex plant genomes like the allopolyploids canola and wheat, SNP array markers can potentially be non-specific in terms of physical position, representing different homoeologous loci in different individuals (Mason et al., 2017; Makhoul et al., 2020). Furthermore, all methods to build haplotype blocks rely on known marker positions along the genome. These positions are obtained from a reference genome and are not necessarily the same in every population or even genotype. Especially if the reference genome is only distantly related. In such cases, a lack of precision in assembled haplotype blocks and their corresponding haplotypes may limit their potential in genomic prediction. Furthermore, haplotype block borders are not necessarily the same across populations and generations. Even though, Gabriel et al. (2002) showed high harmony of block structure across different human populations, however in plant breeding, with selection favoring positive alleles or haplotypes, this could ultimately change. Especially LD based haplotype blocks may only be useful for very few generations, since initially defined blocks will rapidly be disrupted by recombination or extended due to selection in later generations as the breeding program progresses. Indeed, an important goal of breeding is to accumulate favorable alleles through selection and recombination. This underlines the need for constant updating of both, the haplotype block assignment and the prediction model. Furthermore, besides the two fixed window approaches, all of the methods tested are only capable of identifying a proxy to true chromosomal recombination breakpoints. Even though crossovers tends to aggregate in recombination hotspots (Li and Stephens, 2003; Myers et al., 2006), haplotype blocking methods with limited marker density and population size may not necessarily be able to detect these hotspots. Therefore, there is a need to develop enhanced haplotype blocking pipelines that can effectively capture natural recombination patterns and address challenges associated with polyploidy, structural variations, and chromosomal rearrangements commonly observed in crop plants (Mason et al., 2017; Schiessl et al., 2019). Consequently, ongoing efforts focus on the development of innovative methods to capture local epistatic effects (Pook et al., 2020).

Unfortunately, we could not identify a single optimal haplotype blocking method that suits all datasets. Therefore, it is important to consider haplotype block construction methods and parameters as hyperparameters that require careful optimization, rather than fixed biological parameters. A breeding program that adopts haplotype block-based genomic selection should explore multiple haplotype blocking methods with different parameter settings. In general, the selected method should effectively capture relationships among individuals. Additionally, it is worth examining blocks of large size, as, in the case of the wheat dataset, larger blocks proved beneficial in improving prediction accuracy. The wheat dataset, which had the lowest marker density, generally showed the greatest improvements. This suggests that haplotype block-based genomic selection could be particularly valuable for breeding programs lacking access to high-density SNP arrays. However, further investigation is required in other datasets with varying SNP densities to validate these findings.

Although we observed only marginal beneficial effects of haplotype blocks in the canola, maize and soybean datasets on genomic prediction, they can still have a beneficial effect when used in other contexts. For example, haplotype blocks can help to identify regions of interest for the identification of candidate genes near significant marker-trait associations, or to compare different genotype groups at such loci (Clark, 2004; Li et al., 2017; Vollrath et al., 2021). Moreover, even if the majority of SNP markers exhibit intermediate minor allele frequency in a population, specific combinations of alleles represented as haplotypes may not be common in a population. Therefore, haplotypes can assist in identifying rare variants that have a potential impact on phenotypic traits. (Bloom et al., 2019; Wainschtein et al., 2022; Wang et al., 2023). Furthermore, especially in highly quantitative traits like yield where markers tend to have very small effects on traits, haplotype blocks can identify positive or negative chromosomal segments. This information can be implemented for cross designs to recombine haplotypes with positive effects (Bernardo and Thompson, 2016; Werner et al., 2018a). This can be considerably easier than selecting for single positive SNPs, as their positive effect can be obscured by deleterious SNPs in proximity that are only rarely separated by recombination in subsequent generations.

## 5 Conclusion

As anticipated based on numerous previous reports, our study confirms that haplotype blocks have the potential to enhance genomic selection, although the magnitude of improvement is sometimes only marginal. Haplotype blocks can particularly compensate for model differences when there is considerable variation in model performance across different prediction models. The extent of improvement with haplotypes compared to SNP-based predictions seem to be highly dependent on factors such as population, population structure, trait, and model. for a multitude of different traits from different crop species with different genome properties and breeding schemes, we were unable to identify optimal methods or parameters for constructing haplotype blocks in terms of prediction accuracy. Approaches based on LD resulted in improved prediction accuracies across various traits and demonstrated robustness in LD-threshold selection. However, the greatest improvements were observed with haplotype blocks consisting of entire chromosomes. Therefore, we recommend treating haplotype block definition as a tunable hyperparameter when employing genomic selection, taking into account extremely large haplotype blocks.

## Data availability statement

Publicly available datasets were analyzed in this study. This data can be found here: Please refer to the original publications of the four datasets.

## Author contributions

SW and RS designed the study. SW conceived the analysis, MF developed the software for Linkage Disequilibrium (LD) based haplotyping. KV-F and MF supervised the statistical analysis. SW wrote the manuscript. RS and KV-F revised the manuscript. All authors contributed to the article and approved the submitted version.
