# Supplementary material for: Haplotype blocks for genomic prediction: a comparative evaluation in multiple crop datasets
Source: Front Plant Sci. 2023 Sep 5;14:1217589. doi: 10.3389/fpls.2023.1217589 (PMC10507710; doi:10.3389/fpls.2023.1217589)
Supplement: Supplementary file 1 [file DataSheet_1.docx]

Supplementary Material

**Haplotype blocks for genomic prediction: A comparative evaluation in multiple crop datasets**

Sven E. Weber^1*^, Matthias Frisch^2^, Rod J. Snowdon^1^, Kai P. Voss-Fels^3^

*** Correspondence:**

Sven E. Weber
Sven.E.Weber@agrar.uni-giessen.de

## Supplementary Figures

**
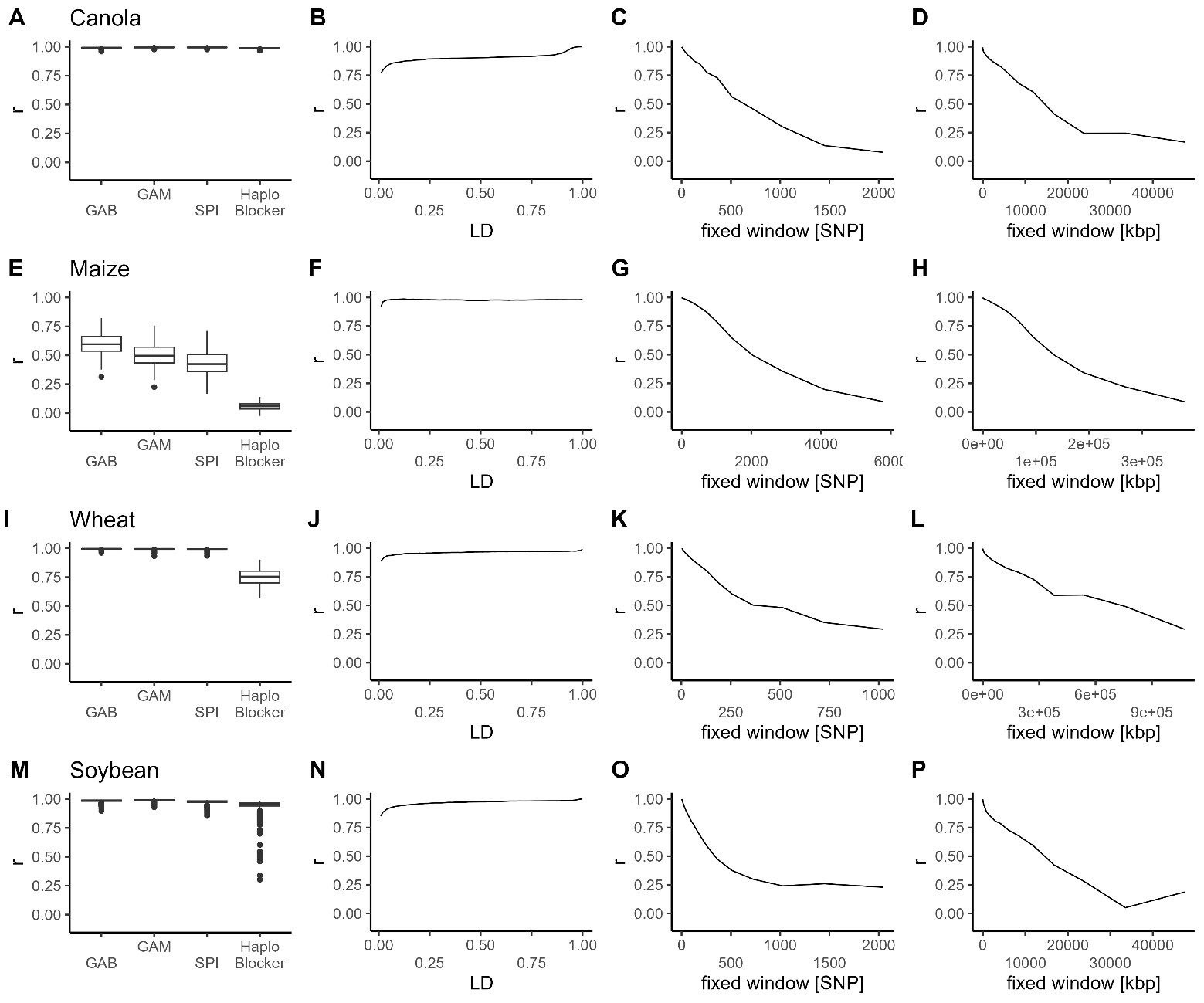
**

**Figure S1** Mean Correlation (r) between SNP and haplotype based genomic relationship coefficients identified by the methods implemented in “Haploview” and “HaploBlocker” (**A**, **E**, **I**, **M**), LD (**B**, **F**, **J**, **N**), fixed window of adjacent base pairs (**C**, **G**, **K**, **O**) and fixed window of adjacent markers (**D**, **H**, **L**, **P**) based haplotype blocks, in canola (**A**, **B**, **C**, **D**), maize (**E**, **F**, **G**, **H**), wheat (**I**, **J**, **K**, **L**) and soybean (**M**, **N**, **O**, **P**)


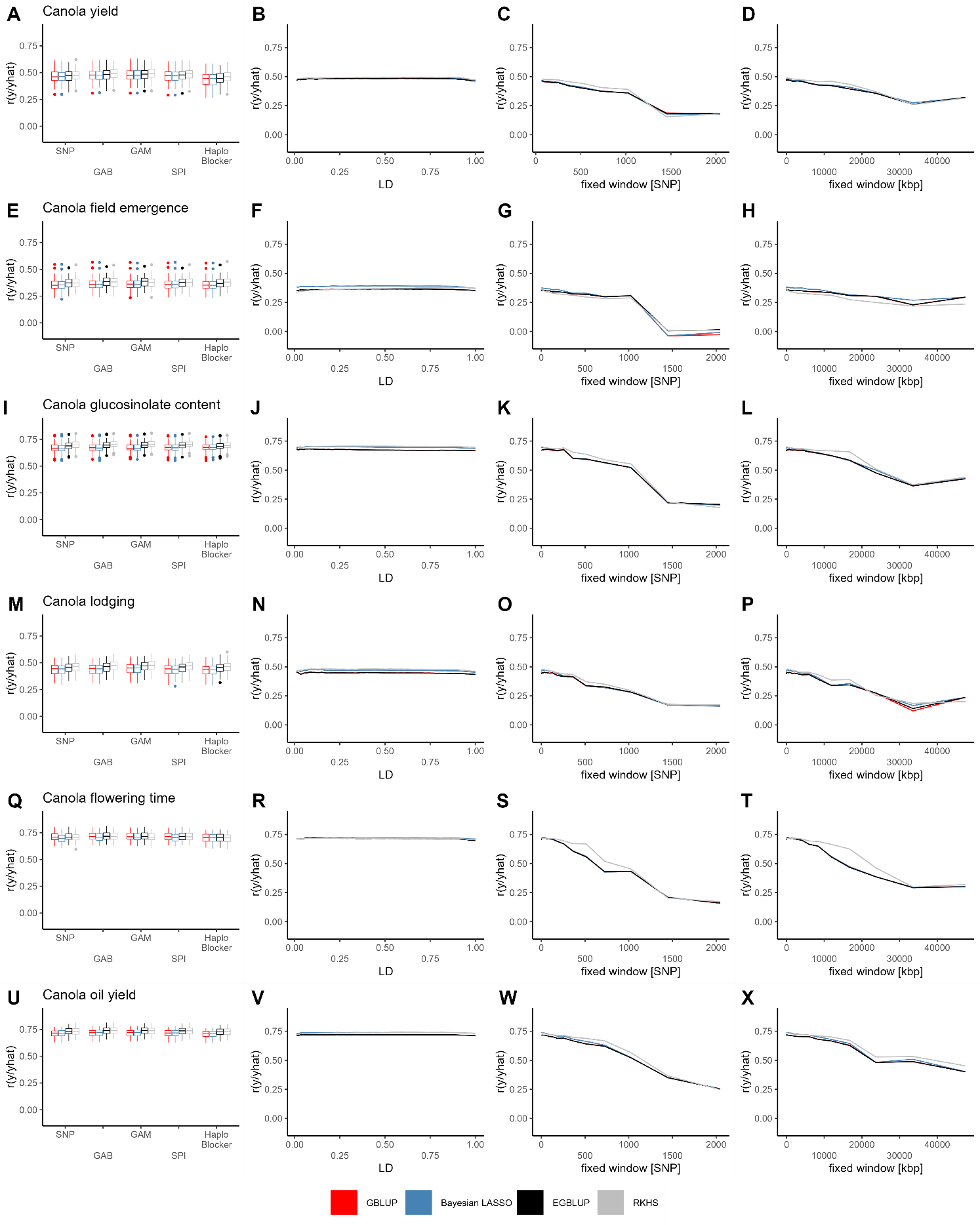
**Figure S2** Prediction accuracy (r) of GBLUP (red), Bayesian LASSO (blue), EGBLUP (black) and RKHS (grey) with SNPs,“Haploview” and “HaploBlocker” (**A**, **E**, **I**, **M**, **Q**, **U**), LD (**B**, **F**, **J**, **N**, **R**, **V**), fixed window of adjacent base pairs (**C**, **G**, **K**, **O**, **S**, **W**) and fixed window of adjacent markers (**D**, **H**, **L**, **P**, **T**, **X**) based haplotype blocks, in canola: seed yield (**A**, **B**, **C**, **D**), field emergence (**E**, **F**, **G**, **H**), glucosinolate content (**I**, **J**, **K** ,**L**),lodging (**M**, **N**, **O**, **P**), flowering time (**Q**, **R**, **S**, **T**), oil yield (**U**, **V**, **W**, **X**). Individual points in the lines represent the mean over all cross validation runs for each haplotype block parameter and model combination


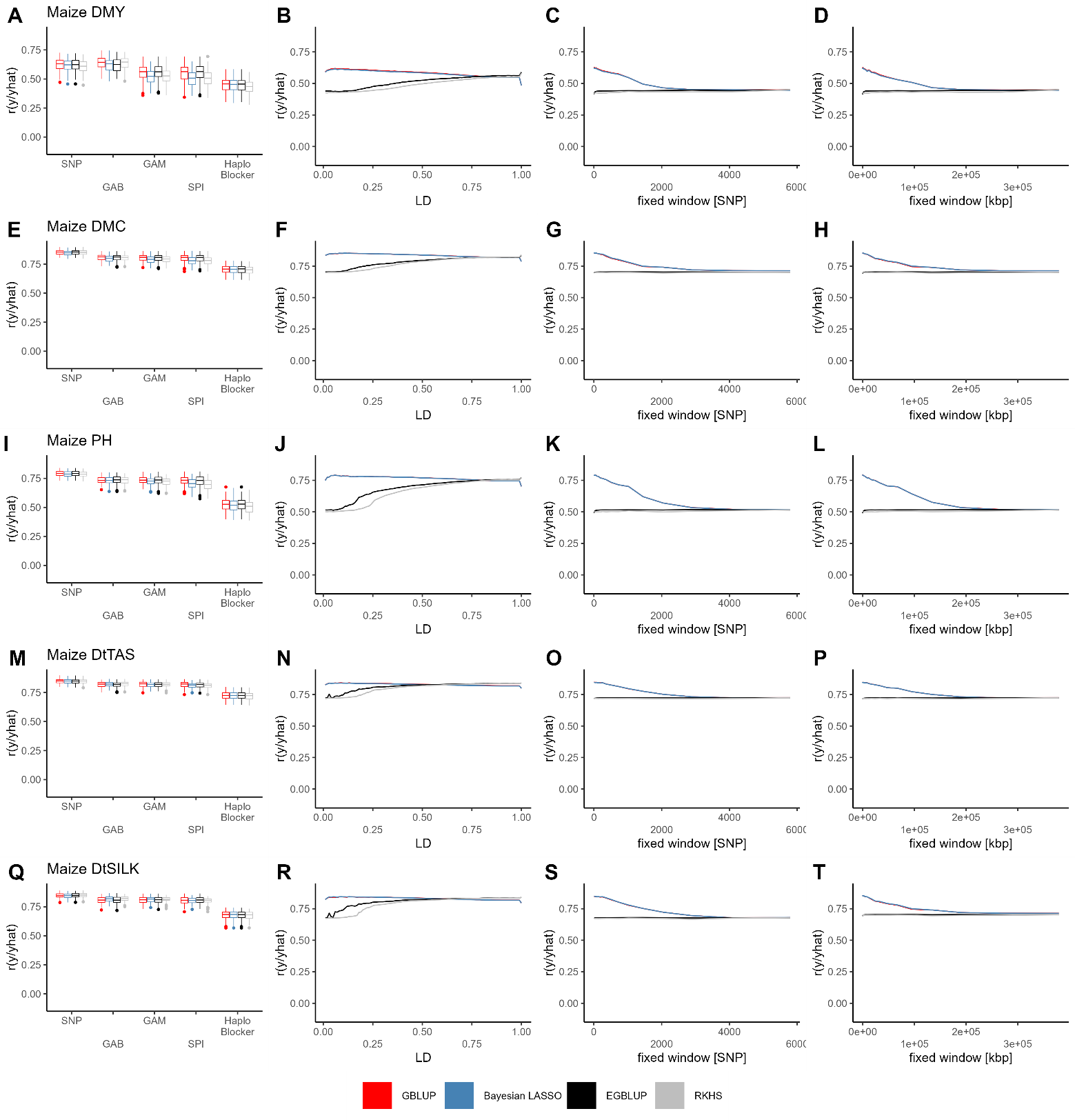
**Figure S3** Prediction accuracy (r) (random cross validation) of GBLUP (red), Bayesian LASSO (blue), EGBLUP (black) and RKHS (grey) with SNPs,*“Haploview”* and *“HaploBlocker”* (**A, E, I, M, Q**), LD (**B, F, J, N, R**), fixed window of adjacent base pairs (**C, G, K, O, S**) and fixed window of adjacent markers (**D, H, L, P, T**) based haplotype blocks, in maize: DMY (**A, B, C, D**), DMC (**E, F, G, H**), PH (**I, J, K, L**), DtTAS (**M, N, O, P**), DtSILK (**Q, R, S, T**). Individual points in the lines represent the mean over all cross validation runs for each haplotype block parameter and model combination


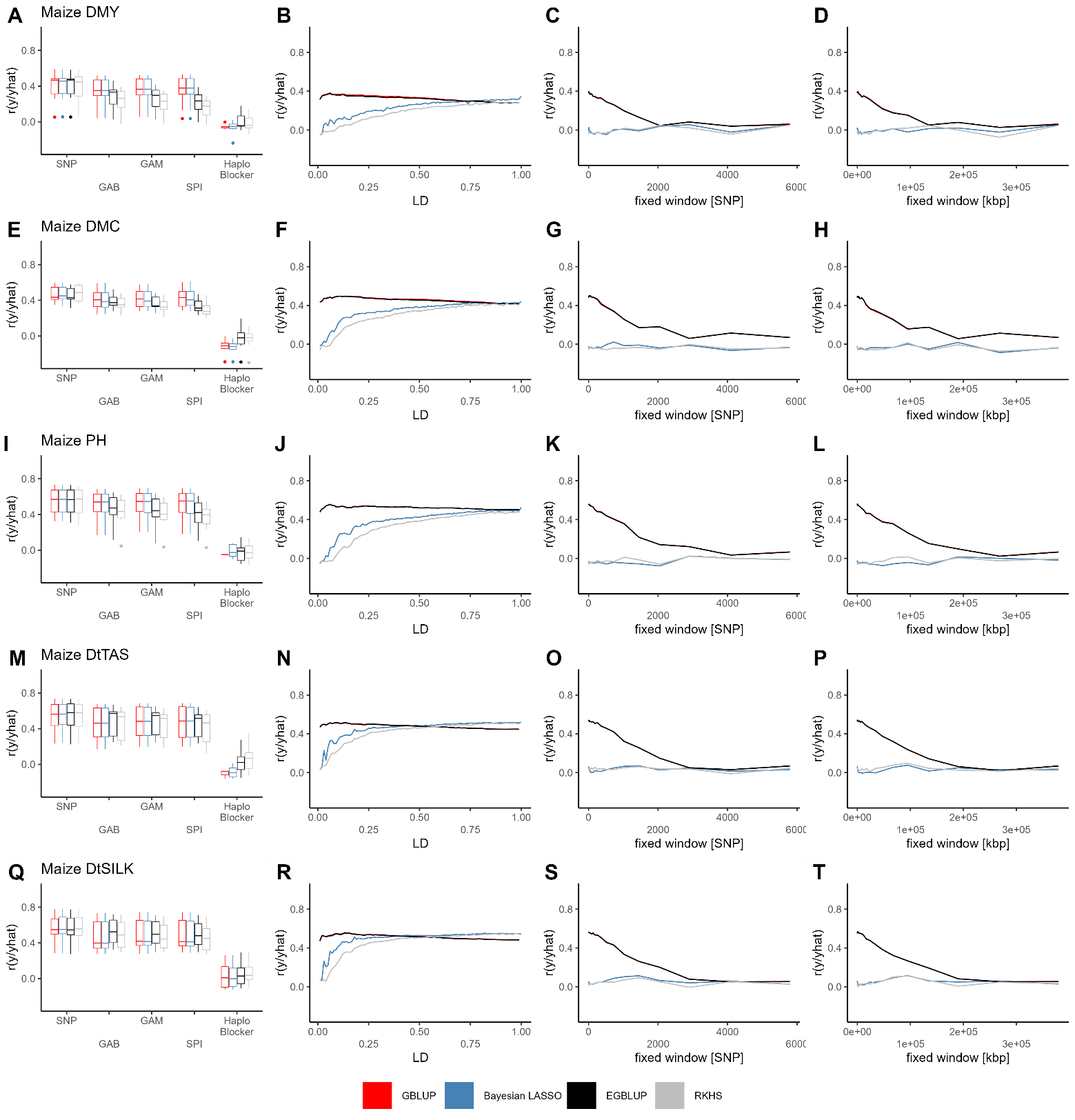
**Figure S4** Prediction accuracy (r) (family-wise cross validation) of GBLUP (red), Bayesian LASSO (blue), EGBLUP (black) and RKHS (grey) with SNPs,*“Haploview”* and *“HaploBlocker”* (**A, E, I, M, Q**), LD (**B, F, J, N, R**), fixed window of adjacent base pairs (**C, G, K, O, S**) and fixed window of adjacent markers (**D, H, L, P, T**) based haplotype blocks, in maize: DMY (**A, B, C, D**), DMC (**E, F, G, H**), PH (**I, J, K, L**), DtTAS (**M, N, O, P**), DtSILK (**Q, R, S, T**). Individual points in the lines represent the mean over all cross validation runs for each haplotype block parameter and model combination


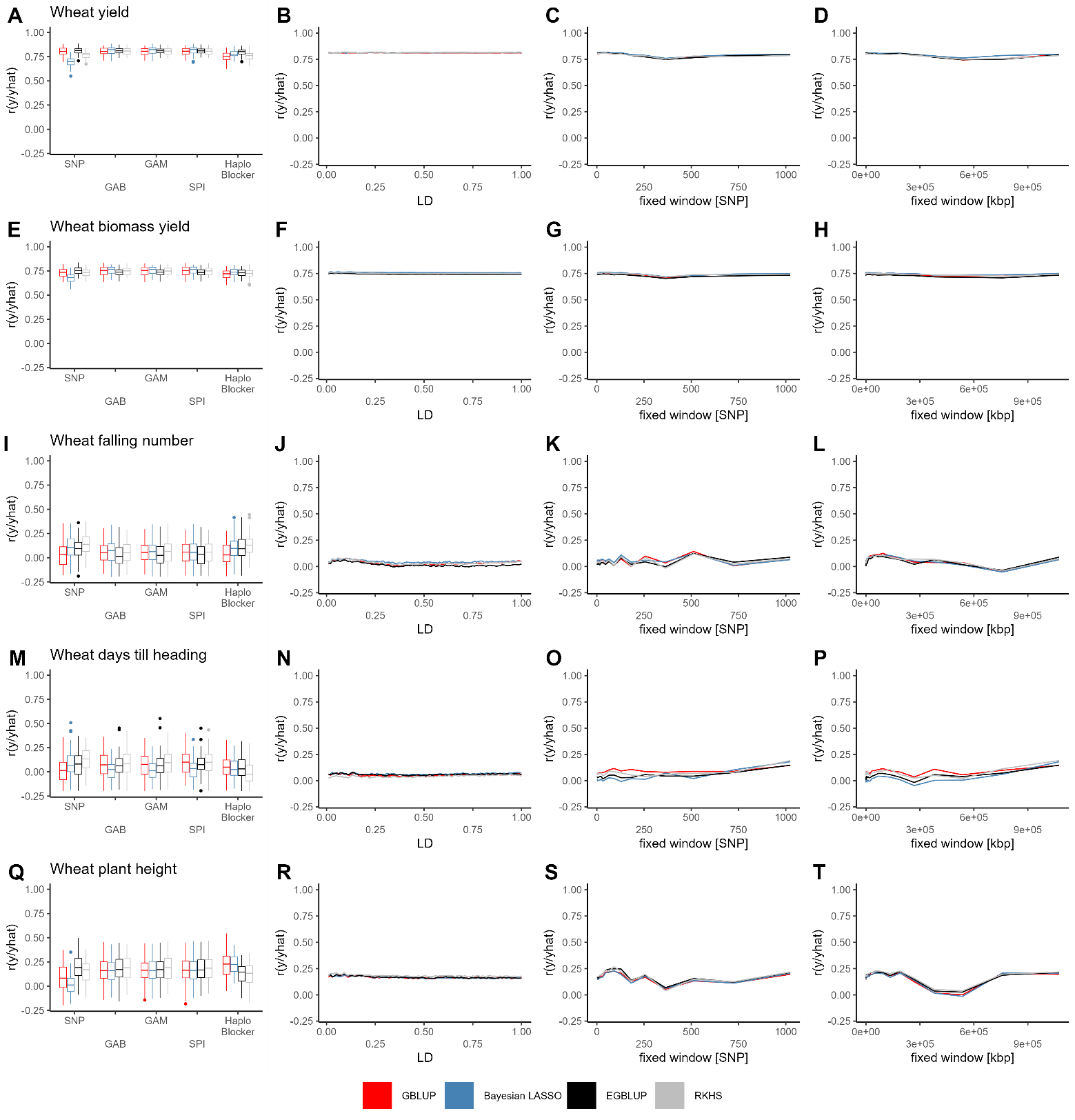
**Figure S5** Prediction accuracy (r) of GBLUP (red), Bayesian LASSO (blue), EGBLUP (black) and RKHS (grey) with SNPs,*“Haploview”* and *“HaploBlocker”* (**A, E, I, M, Q**), LD (**B, F, J, N, R**), fixed window of adjacent base pairs (**C, G, K, O, S**) and fixed window of adjacent markers (**D, H, L, P, T**) based haplotype blocks, in wheat: seed yield (**A, B, C, D**), biomass yield (**E, F, G, H** ), falling number (**I, J, K, L**), days till heading (**M, N, O, P**), plant height (**Q, R, S, T**). Individual points in the lines represent the mean over all cross validation runs for each haplotype block parameter and model combination


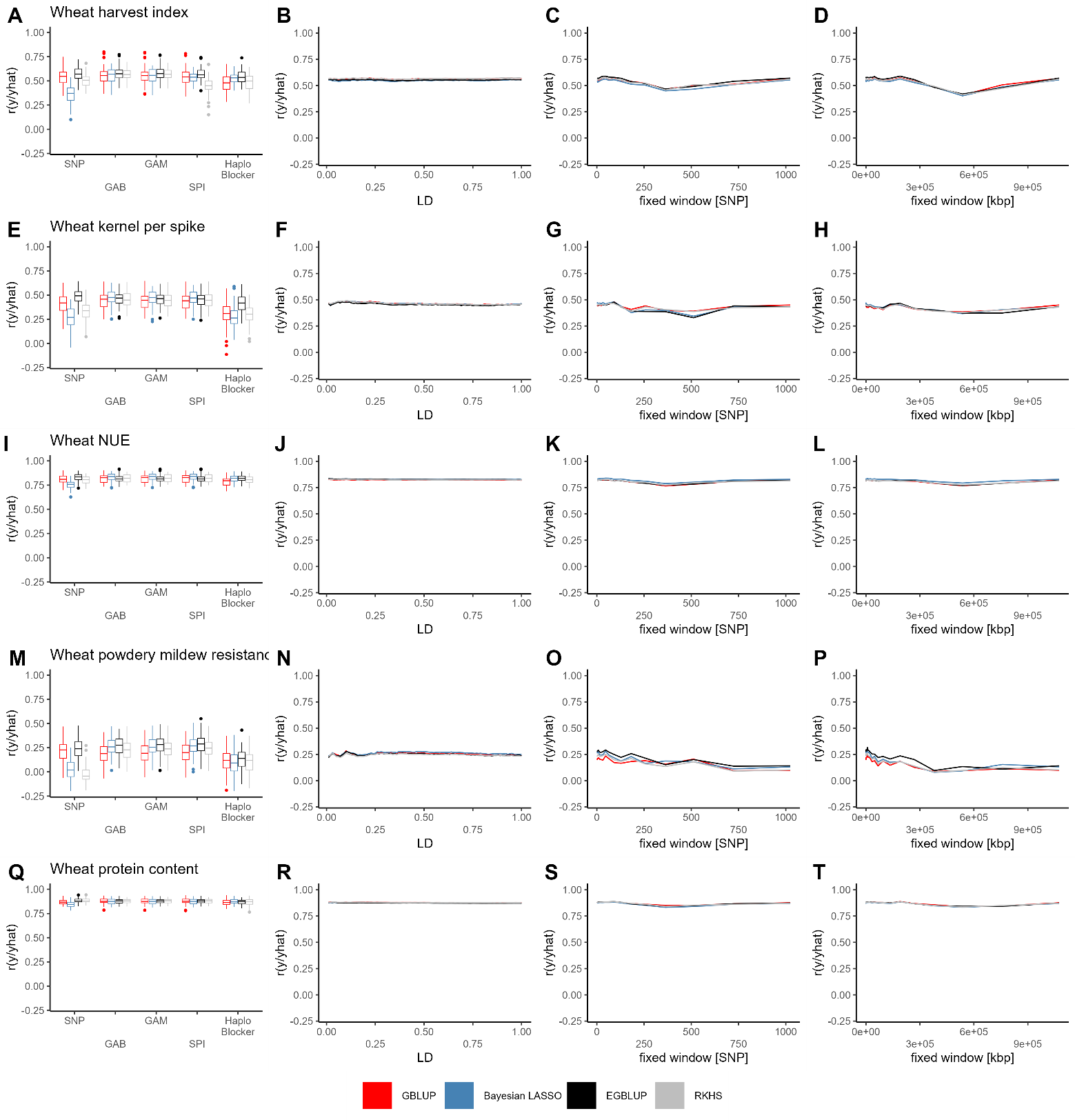
**Figure S6** Prediction accuracy (r) of GBLUP (red), Bayesian LASSO (blue), EGBLUP (black) and RKHS (grey) with SNPs*,“Haploview”* and *“HaploBlocker”* (**A, E, I, M, Q**), LD (**B, F, J, N, R**), fixed window of adjacent base pairs (**C, G, K, O, S**) and fixed window of adjacent markers (**D, H, L, P, T**) based haplotype blocks, in wheat: harvest index (**A, B, C, D**), kernel spike^-1^ (**E, F, G, H** ), NUE (**I, J, K, L**), powdery mildew resistance (**M, N, O, P**), protein content (**Q, R, S, T**). Individual points in the lines represent the mean over all cross validation runs for each haplotype block parameter and model combination


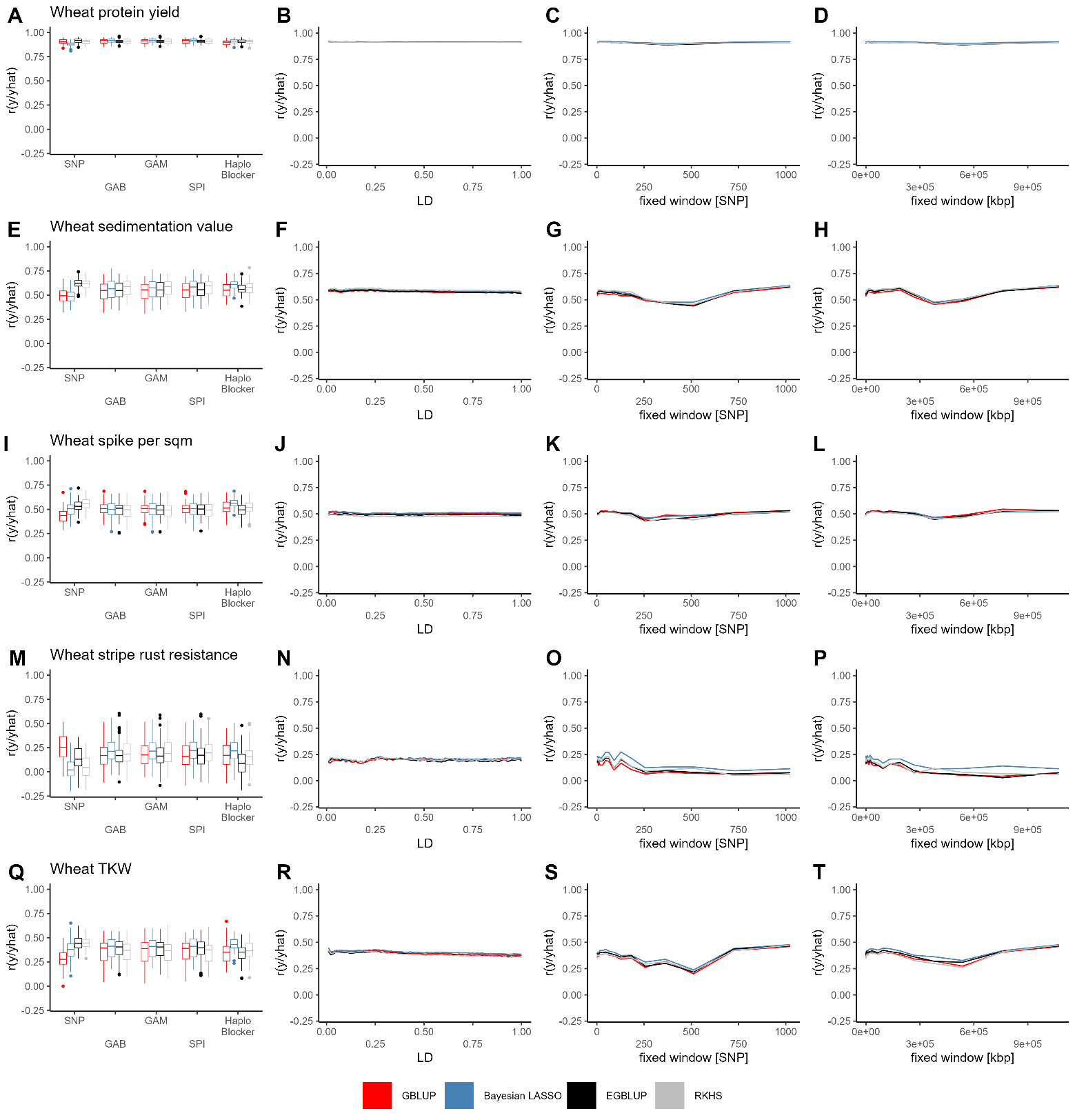
**Figure S6** Prediction accuracy (r) of GBLUP (red), Bayesian LASSO (blue), EGBLUP (black) and RKHS (grey) with SNPs,*“Haploview”* and *“HaploBlocker”* (**A, E, I, M, Q**), LD (**B, F, J, N, R**), fixed window of adjacent base pairs (**C, G, K, O, S**) and fixed window of adjacent markers (**D, H, L, P, T**) based haplotype blocks, in wheat: protein yield (**A, B, C, D**), sedimentation value (**E, F, G, H** ), spike m^-2^ (**I, J, K, L**), stripe rust resistance (**M, N, O, P**), TKW (**Q, R, S, T**). Individual points in the lines represent the mean over all cross validation run for each haplotype block parameter and model combination


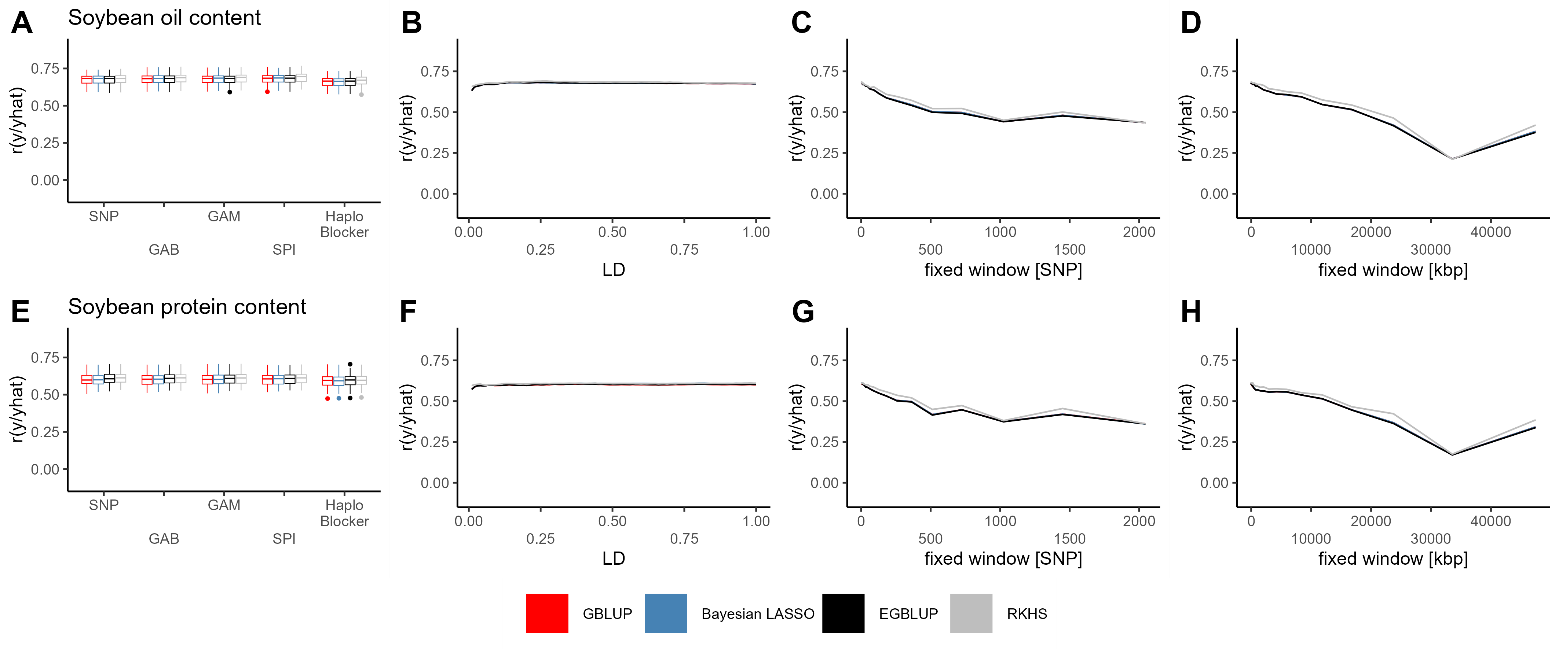
**Figure S7** Prediction accuracy (r) of GBLUP (red), Bayesian LASSO (blue), EGBLUP (black) and RKHS (grey) with SNPs,*“Haploview”* and *“HaploBlocker”* (**A, E**), LD (**B, F**), fixed window of adjacent base pairs (**C, G**) and fixed window of adjacent markers (**D, H**) based haplotype blocks, in soybean: oil content (**A, B, C, D**), protein content (**E, F, G, H** ). Individual points in the lines represent the mean over all cross validation run for each haplotype block parameter and model combination
